# Unusual Oral Presentations of Herpes Simplex Virus‐1: A Series of Eight Cases and Literature Review

**DOI:** 10.1111/scd.70129

**Published:** 2025-12-18

**Authors:** João Paulo Gonçalves de Paiva, Sebastião Silvério Sousa‐Neto, Maurilo Antônio Correia Humberto, Marco Antônio de Oliveira Magalhães, Justin Bubola, José Narciso Rosa Assunção Júnior, Marcelo Marcucci, Marcondes Sena‐Filho, Celso Augusto Lemos, Cristina Saldivia‐Siracusa, Alan Roger Santos‐Silva, Jacks Jorge, Pablo Agustin Vargas

**Affiliations:** ^1^ Oral Diagnosis Department Faculdade De Odontologia de Piracicaba Universidade Estadual de Campinas (FOP‐UNICAMP) Piracicaba São Paulo Brazil; ^2^ Oral Medicine in Private Practice Pirassununga São Paulo Brazil; ^3^ Oral Pathology & Oral Medicine Faculty of Dentistry University of Toronto Toronto Ontario Canada; ^4^ Department of Dental and Maxillofacial Sciences Sunnybrook Health Sciences Centre Toronto Ontario Canada; ^5^ Serviço De Estomatologia Hospital Santa Casa Da Misericórdia de Santos. Serviço de Estomatologia, Hospital Infantil Gonzaga Santos São Paulo Brazil; ^6^ Stomatology and Oral and Maxillofacial Surgery Center, Hospital Heliópolis, São Paulo São Paulo Brazil; ^7^ Oral Surgery and Oral Medicine in Private Practice Goiânia Goiás Brazil; ^8^ Departament of Oral Medicine School of Dentistry University of São Paulo São Paulo Brazil

**Keywords:** herpes simplex virus 1, herpetic stomatitis, immunocompetence, mouth diseases, viral diseases

## Abstract

**Aims:**

The aim of this study is to characterize the clinical and microscopic features of recurrent oral/labial HSV‐1 infection with unusual clinical presentations.

**Methods:**

A literature review and retrospective case series of patients with recurrent oral/labial HSV‐1 with unusual presentations were conducted. Data were collected from medical records, and all cases were confirmed by histopathology and immunohistochemistry.

**Results:**

Eight patients (five males, 62.5%; three females, 37.5%) with a mean age of 61.8 years presented with unusual HSV‐1 manifestations, including solitary lesions, white plaque‐like appearance, non‐keratinized site involvement, or prolonged duration. Lesions involved the tongue border (*n* = 4, 50%), anterior ventral tongue, hard palate, posterior ridge crest, and lower lip (*n* = 1, 12.5% each). Presented as well‐demarcated solitary ulcers (*n* = 6, 75%), solitary papule (*n* = 1, 12.5%), and whitish plaque‐like lesions (*n* = 1, 12.5%). Only two patients were immunosuppressed. All cases responded successfully to management, including pharmacotherapy, surgical excision, or photobiomodulation. Unusual clinical presentations of oral/labial HSV‐1 infection are scarcely reported in the literature.

**Conclusion:**

Unusual HSV‐1 manifestations can occur in immunocompetent individuals; however, exclusion of undiagnosed immunosuppression remains necessary. A definitive diagnosis relies on careful histopathological evaluation of ulcer edges, which is crucial for guiding appropriate treatment. Further documentation of similar cases is essential to reinforce that these unusual presentations can indeed affect immunocompetent patients.

## 1 Introduction

Human herpes simplex virus type 1 (HSV‐1) is highly prevalent worldwide, with the highest rates being reported in developing countries [1, 2, 3, 4, 5, 6]. The transmission occurs primarily through direct contact with oral secretions or saliva from infected individuals, regardless of symptomatic status [3, 5, 7, 8]. The clinical course of oral HSV‐1 infection typically begins with primary infection in childhood, progressing to lifelong latency in the trigeminal ganglion. During this latent period, recurrent viral activations can cause multiple small vesicles/ulcers on the keratinized tissues of the hard palate and gingiva, which typically heal within about two weeks [1, 2, 3].

In immunocompetent individuals, primary and recurrent oral HSV‐1 infections are generally asymptomatic or manifest with mild‐to‐moderate symptoms, with lesions predominantly affecting keratinized mucosa [7, 9, 10]. Conversely, in immunocompromised patients, herpetic infection often exhibits atypical features, including extensive, aggressive, slow‐healing, and markedly painful lesions [7, 8, 11]. These unusual lesions may mimic other conditions, such as mucositis, drug‐induced reactions, neutropenic ulcers, or other viral infections (e.g., cytomegalovirus and herpangina), complicating diagnosis and necessitating adjunct investigations such as viral culture, polymerase chain reaction (PCR), Tzanck smear, or HSV antibody serum testing [10, 12].

In the absence of clear clinical signs of HSV‐1 infection, standard diagnostic tests are often not performed, and consequently, incisional biopsy may serve as the initial diagnostic step. Histopathological analysis of biopsied tissue may reveal characteristic cytological changes, including multinucleation and glassy nuclear viral inclusions [13]. Immunohistochemical (IHC) evaluation may be another valuable diagnostic tool, especially in cases with nonspecific morphology or scarcity of virally altered cells [13, 14].

While immunosuppressed individuals exhibit heightened susceptibility to HSV‐1 and an increased likelihood of atypical presentations, systematic documentation of those cases of unusual HSV‐1 remains sparse [10, 11, 15, 16, 17, 18, 19, 20, 21, 22, 23, 24, 25, 26, 27, 28, 29]. This knowledge gap hinders a comprehensive understanding of the interplay between immunological status and unusual HSV‐1 infections. To address this, the present study characterizes IHC‐confirmed recurrent oral and labial cases in patients with and without apparent immunosuppression, aiming to clarify the clinicopathological correlations and improve the diagnostic recognition of these uncommon presentations.

## 2 Material and Methods

### 2.1 Study Design and Patient Selection

This retrospective descriptive case series analyzed patients diagnosed with recurrent oral or labial herpes who presented with unusual clinical features at a Brazilian Oral Pathology Laboratory and a Canadian Oral Pathology Service between 2015 and 2025. This study was conducted in accordance with the recommendations of the Declaration of Helsinki and was approved by the Research Ethics Committee of the University. The privacy rights of human subjects have been observed, and informed consent was obtained from all participants involved in the study.

Inclusion criteria comprised patients of both sexes, over 18 years old, with or without apparent immunosuppression, presenting with unusual oral or labial HSV‐1 infections confirmed by histopathology and IHC. We defined a clinical manifestation as unusual if it diverged from the classic presentation of oral HSV‐1. This included solitary lesions, involvement of non‐keratinized mucosa, prominent painful symptoms, an absence of typical small ulcers or vesicles or a duration exceeding 2 weeks. Cases without histopathological or IHC confirmation were excluded. Clinical and laboratory data were retrospectively collected from medical records and anonymized.

A detailed description of the patient cohort, including demographics and clinical presentation, is provided in the Results section.

### 2.2 Histological and Immunohistochemical Analyses

Formalin‐fixed, paraffin‐embedded tissue sections were stained with hematoxylin and eosin (H&E) for morphological assessment. IHC detection of HSV‐1 was performed following heat‐induced epitope retrieval in citrate buffer (pH 6), peroxidase blocking, and incubation with an anti‐HSV‐1 monoclonal antibody (B0114, Dako; 1:100). Detection employed the MACH4 Universal HRP Polymer system (Biocare Medical) and DAB Metal Enhanced Substrate (Thermo Fisher Scientific), with hematoxylin counterstaining. Photomicrographs were acquired using a Leica DM5000 microscope with a DMR camera at 10x, 20x, and 40x magnifications.

### 2.3 Literature Review

A narrative review was conducted through PubMed, SciELO, and LILACS databases using the following search strategy: (Herpes Simplex OR herpes simplex OR HSV OR herpesvirus) AND (oral OR mouth OR labial OR mucosa OR tongue OR gingiva OR palate OR buccal OR orofacial OR stomatitis) AND (atypical OR unusual OR rare OR aberrant OR variant OR differential diagnosis OR misdiagnosed OR mimicking) AND (immunocompromised OR immunosuppressed OR HIV OR AIDS OR cancer OR chemotherapy OR transplant OR immunodeficiency). Case reports and case series published in English or Portuguese were included without time restrictions. Study selection and data extraction were independently performed by two researchers (J.P.G.P. and S.S.S.N.), with disagreements resolved by a third reviewer (P.A.V.). Extracted variables included patient demographics, lesion site, clinical presentation, comorbidities, treatments, outcomes, and follow‐up.

## 3 Results

### 3.1 Clinical Features and Treatment Outcomes

**FIGURE 1:**
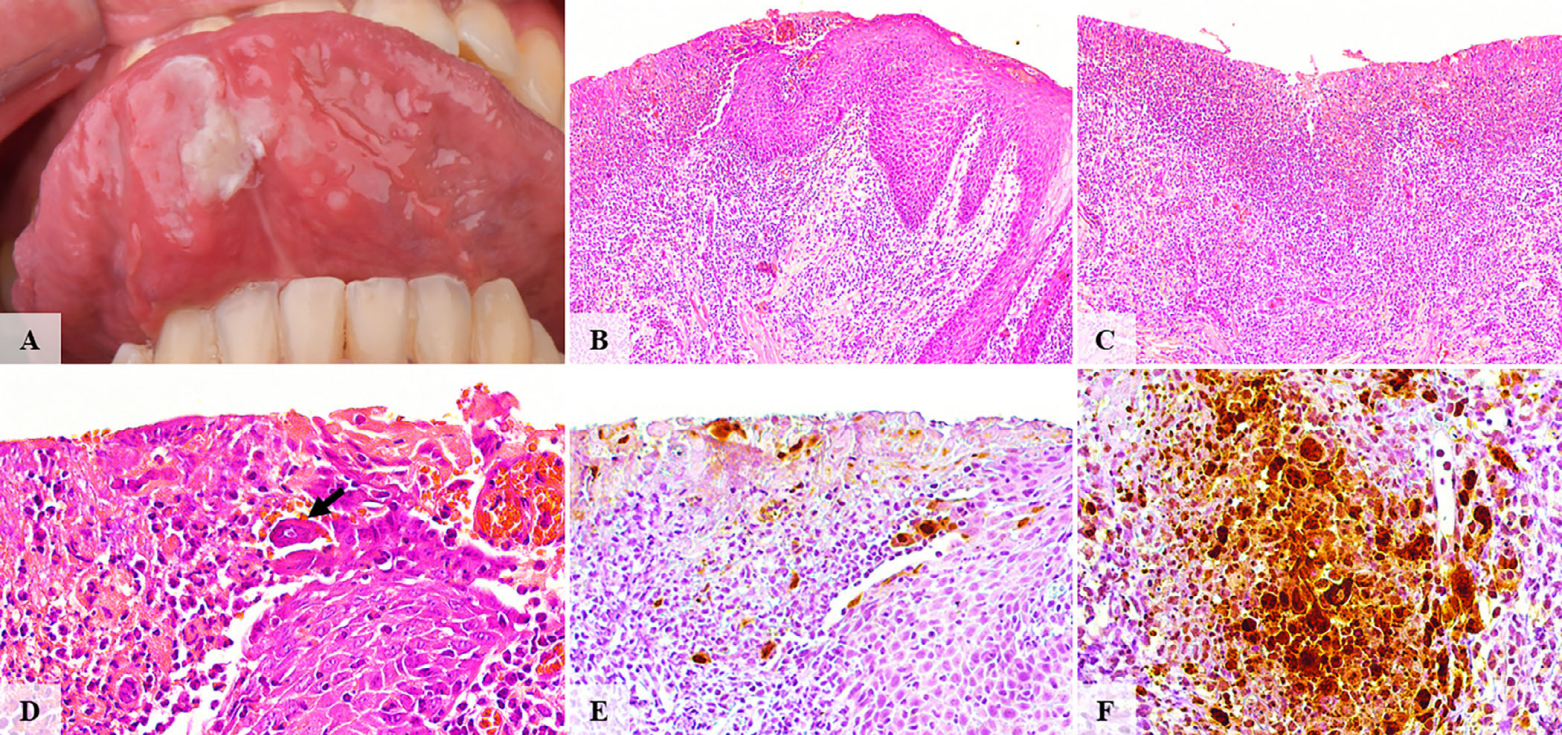
Clinical, histopathological, and immunohistochemical features of case #1. (A) An extensive ulcerative lesion coated by a whitish pseudomembrane involving the ventral surface of the anterior tongue. (B) A well‐demarcated ulcer. (C) Ulcerated surface was covered with a fibrinopurulent membrane and surrounded by a dense inflammatory infiltrate composed of lymphocytes and neutrophils. (D) At the transitional zones between intact and ulcerated regions, keratinocytes exhibited cytopathic changes, including nuclear molding and multinucleation. (E,F) HSV‐1 immunohistochemical reaction highlights HSV‐1‐infected keratinocytes.

**FIGURE 2:**
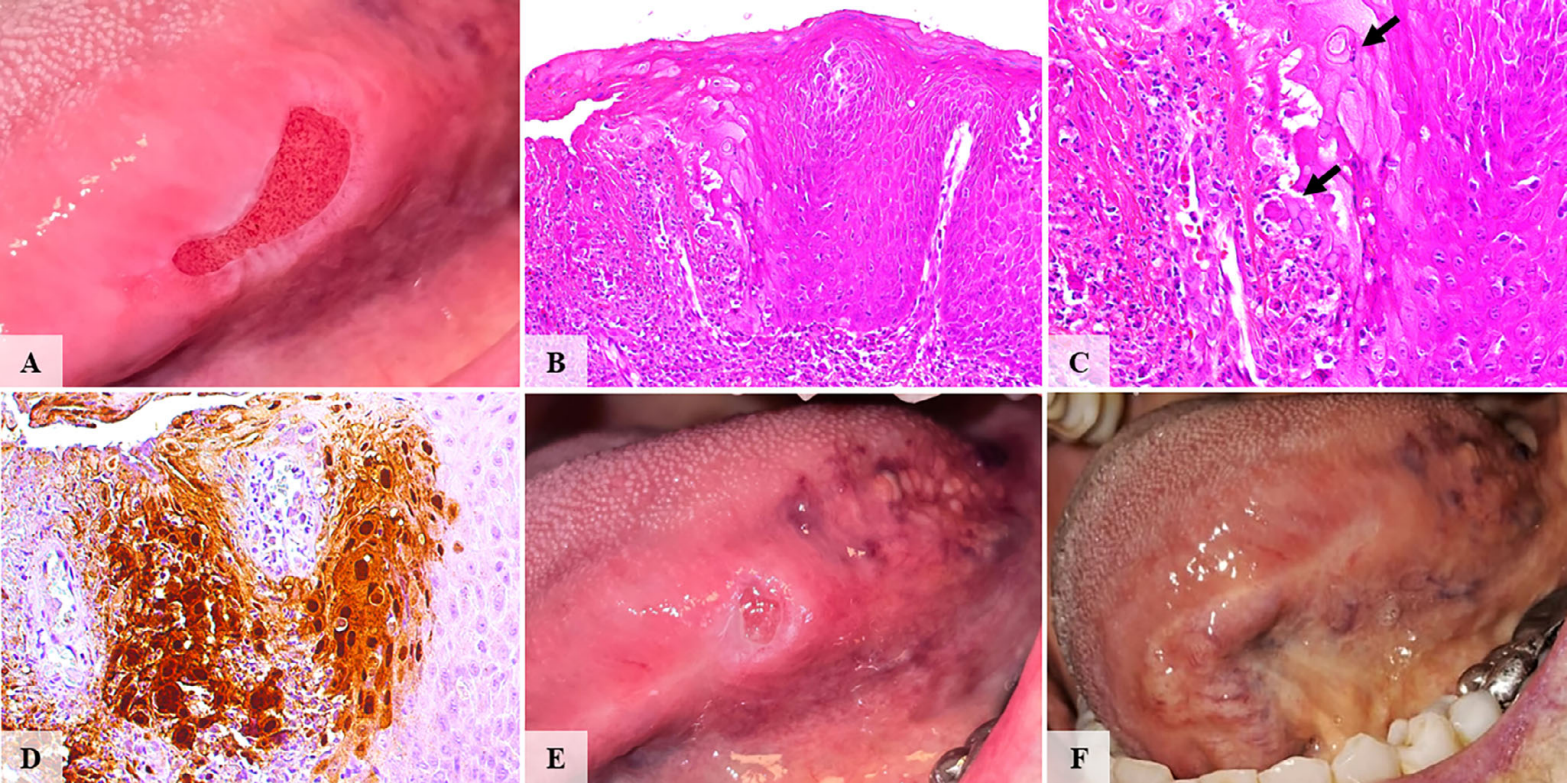
Clinical, histopathological, and immunohistochemical features of case #2. (A) An extensive erythematous ulcer with a whitish border localized to the left posterior border of the tongue. (B) Well‐defined ulcerated area. (C) Altered keratinocytes displaying nuclear molding, multinucleation, and glassy nuclei with viral inclusions. (D) Cytopathically altered keratinocytes exhibited strong HSV‐1 immunoreactivity. (E) The clinical appearance of the lesion after 20 sessions of low‐level laser therapy demonstrated partial resolution. (F) Complete resolution of the lesion following pharmacotherapy.

**FIGURE 3:**
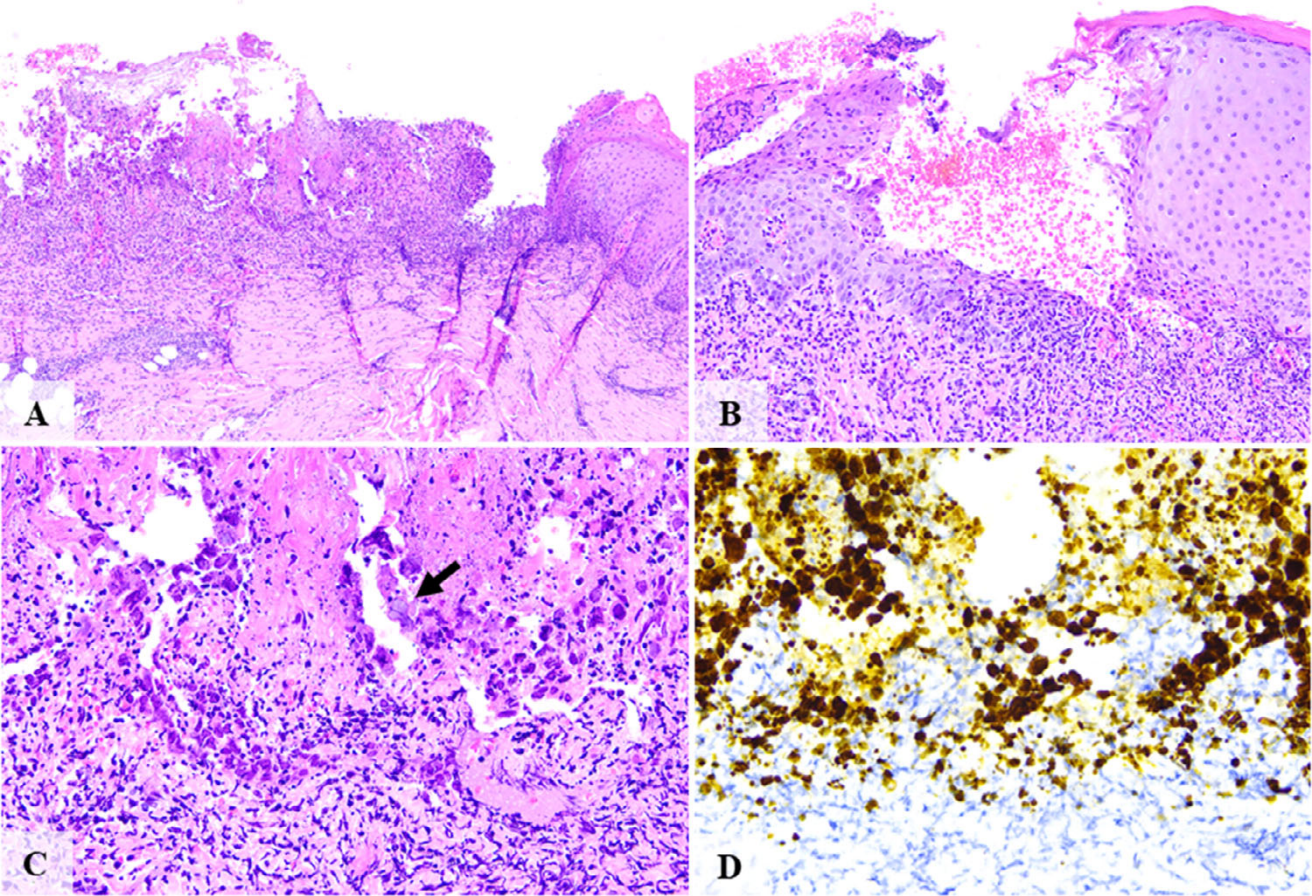
Clinical, histopathological, and immunohistochemical features of case #3. (A, B) A well‐defined ulceration with a dense inflammatory infiltrate. (C) Altered keratinocytes with cytopathic changes. (D) Strong HSV‐1 immunoreactivity in infected keratinocytes.

**FIGURE 4:**
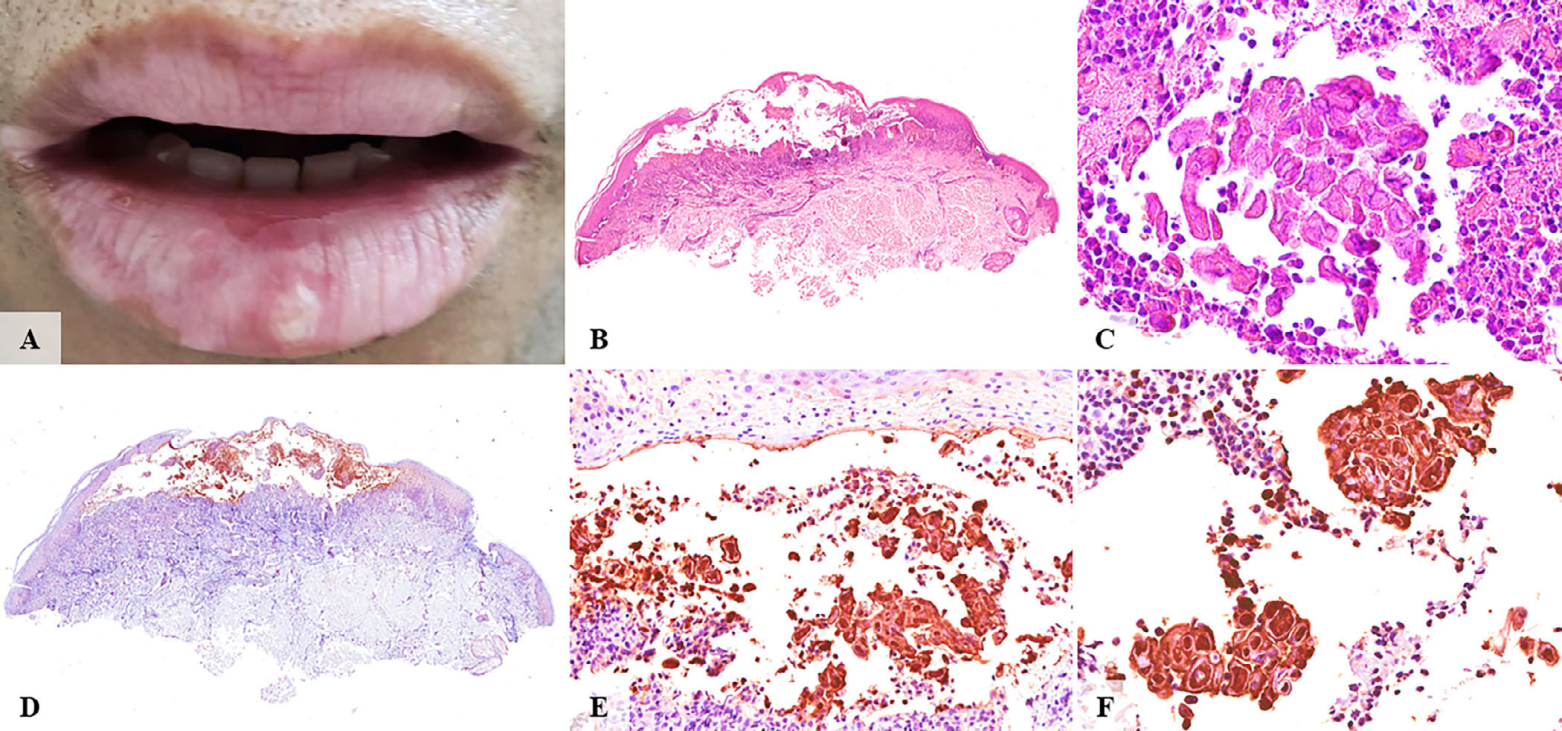
Clinical, histopathological, and immunohistochemical features of case #4. (A) A solitary whitish papule involving the lower lip, accompanied by depigmentation of the vermilion border. (B) A large intraepithelial blister associated with a dense subepithelial inflammatory infiltrate. (C–F) Multiple altered keratinocytes were observed within the large blister. These infected cells exhibited intense immunoreactivity for HSV‐1 immunohistochemical marker.

**FIGURE 5:**
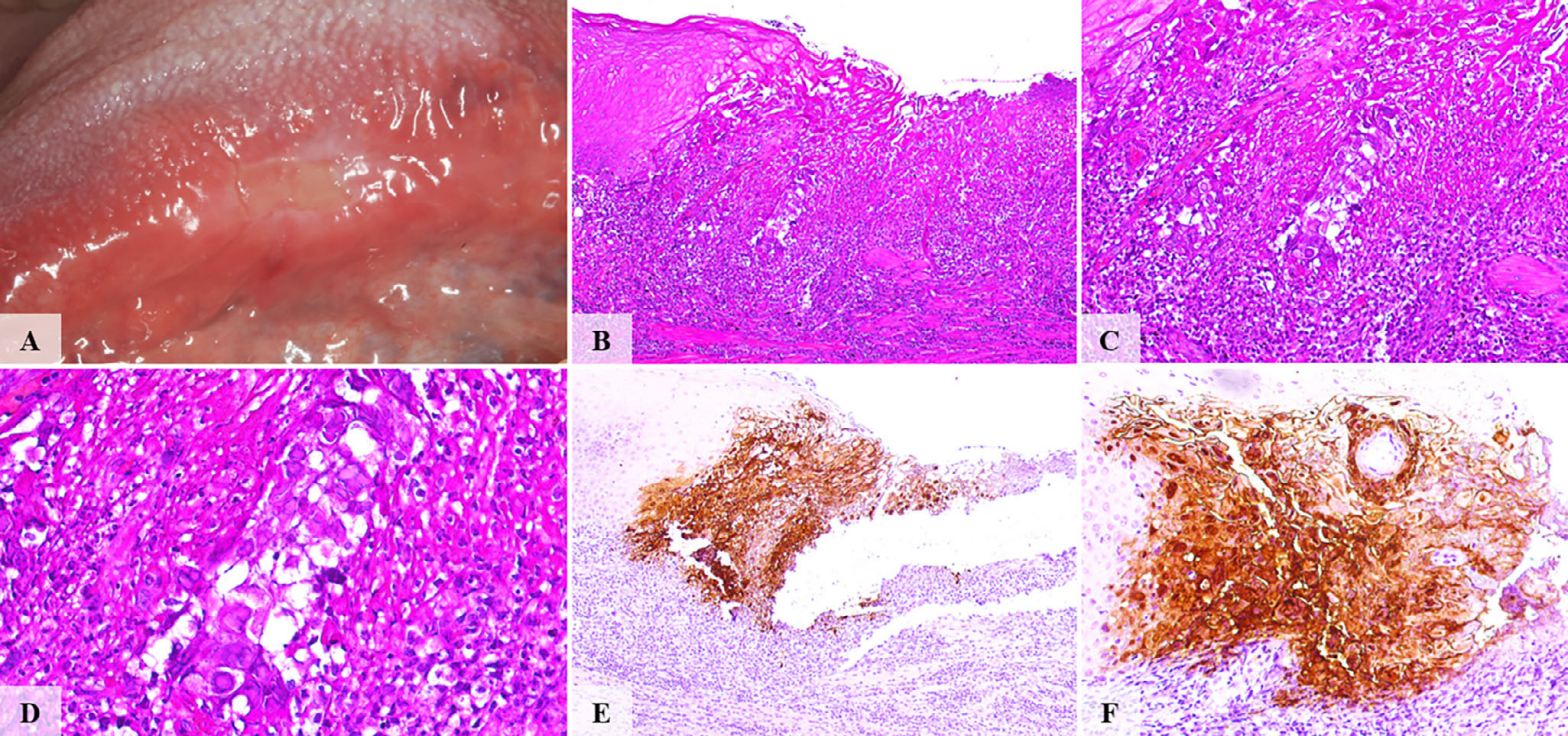
Clinical, histopathological, and immunohistochemical features of case #5. (A) A well‐demarcated, yellowish ulcer involving the left posterior border of the tongue. (B–F) A well‐defined ulceration covered by a fibrinopurulent membrane with an intense mixed inflammatory infiltrate exhibiting numerous HSV‐1‐positive altered multinucleated keratinocytes.

The clinical features and treatment for eight cases of HSV‐1 infection with unusual clinical presentations are described in detail in Table 1. Lesions predominantly affected the tongue border (*n* = 4; 50%), followed by the anterior ventral tongue (*n* = 1; 14.29%), hard palate (*n* = 1; 12.5%), posterior ridge crest (*n* = 1; 12.5%), and lower lip (*n* = 1; 12.5%). Five patients (62.5%) were males, and three (37.5%) were females. Patient ages ranged from 40 to 82 years (mean 61.75 ± 17.18 years, median 67 years). Clinical presentations included well‐demarcated ulcers covered by fibrinopurulent membrane (*n* = 6; 75%), solitary papule (*n* = 1; 12.5%), and whitish plaque‐like lesion (*n* = 1; 12.5%) (Figures 1, 2, 3, 4, 5). Herpes simplex infection was not considered in the initial differential diagnosis for any case. Clinical hypotheses included syphilis or other sexually transmitted infections, immunotherapy‐induced mucositis, vesiculobullous disease, nonspecific chronic ulcer, traumatic ulcerative granuloma with stromal eosinophilia (TUGSE), squamous cell carcinoma, and leukoplakia.

**TABLE 1:** Clinical features of unusual HSV‐1 cases included in this case series study.

Case	Age	Sex	Location	Clinical presentation	Size	Symptoms	Duration	Other diseases/ medications	Clinical hypothesis	Biopsy type	Treatment	Outcome	Follow‐up
#1	40	M	Anterior ventral tongue	Greyish‐to‐whitish ulcer and small vesicles	1 x 1 cm	Intense pain	4 d	No	Syphilis/ other STI	Incisional	Valacyclovir 500 mg – 12h‐12 h for 5 d + Prednisolone 20 mg – 24h‐24 h for 3 d	Total response	LTF
#2	76	F	Left posterior tongue border	Single ulcer with well‐defined borders	2.8 x 1.4 cm	Intense pain	3 month	CM, IMT/ RA, methotrexate	IMT‐induced mucositis	Incisional	Photobiomudulation/ Valacyclovir 500 mg ‐ 12h‐12 h for 5 d	Total response	7 mo‐ FOD
#3	82	M	Right hard palate	Whitish plaque‐like	NA	Asymptomatic	NA	No	Leukoplakia	Incisional	No treatment	NA	NA
#4	41	M	Lower lip	Single soft‐consistency papule	0.4 x 0.3 cm	Asymptomatic	20 d	No	Vesiculobullous disease (NOS)	Excisional	Topic Acyclovir 50 mg/g – 6h‐6 h for 10 d	NA	LTF
#5	75	F	Left posterior tongue border	Single ulcer with well‐defined borders	0.7 x 0.4 cm	Intense pain	3 month	CLL	Traumatic ulcer/ TUGSE	Excisional	Acyclovir 400 mg – 5h‐5 h for 5 d	Total response	1 mo‐ FOD
#6	73	F	Left posterior tongue border	Single ulcer with well‐defined borders	1.4 x 0.8 cm	NA	6 w	NA	Nonspecific chronic ulcer	Excisional	Surgical excision	NA	LTF
#7	61	M	Right tongue border	Single ulcer	0.5 cm	NA	NA	No	Traumatic ulcer/ SCC	Incisional	NA	NA	NA
#8	46	M	Left posterior ridge crest	Single ulcer	1 cm	NA	NA	No	Leukoplakia	Incisional	NA	NA	NA

Abbreviations: CM, cutaneous melanoma; FOD, free of disease; IMT, immunotherapy; LTF, lost to follow‐up; NA, not available; NOS, not otherwise specified; RA, rheumatoid arthritis; SCC, squamous cell carcinoma; STI, sexually transmitted infection; TUGE, traumatic ulcerative granuloma with stromal eosinophilia.

Symptom data and duration were documented for five patients, with three (60%) reporting painful lesions and the remaining two (40%) having asymptomatic lesions, with durations ranging from 4 days to 3 months. Among patients with complete clinical data, only two exhibited an apparent immunocompromised status, which likely contributed to the unusual presentation of herpes simplex infection. These patients (case #2 and case #5) were a 76‐year‐old woman receiving methotrexate therapy for rheumatoid arthritis and immunotherapy (nivolumab, ipilimumab, and tocilizumab) for melanoma, and a 75‐year‐old female patient with chronic lymphocytic leukemia, respectively.

Patient management varied across cases. Cases #1, #4, and #5 received pharmacotherapy (oral or topical antivirals), while case #6 underwent surgical excision alone due to a provisional hypothesis of nonspecific chronic ulcer. Case #3 received no treatment. Case #2 was treated with a combined regimen of photobiomodulation and oral antivirals but achieved total clinical response only after pharmacotherapy was added. The pharmacological treatment led to complete lesion resolution in cases #1 and #5. Cases #4 and #6 were lost to follow‐up, precluding assessment of treatment outcomes. Treatment details, outcomes, and follow‐up data were unavailable for the remaining patients.

### 3.2 Histological and Immunohistochemical Features

The ulcerated surfaces were consistently covered by a fibrinopurulent membrane. All cases showed a dense mixed inflammatory infiltrate of neutrophils and lymphocytes within ulcerated and blistering regions. Variable numbers of virally altered keratinocytes exhibiting multinucleation, nuclear molding, and glassy viral inclusions were observed at the transition zones between ulcerated and intact epithelium, as well as at subjacent ulcer regions and blistering areas (Figures 1, 2, 3, 4, 5).

The altered keratinocytes displayed intense HSV‐1 IHC positivity, confirming transcriptionally active viral DNA, and defining the diagnosis of herpes simplex infection with unusual clinical presentation (Figures 1, 2, 3, 4, 5).

### 3.3 Literature Review

Our literature review on unusual clinical presentations of HSV‐1 infection affecting the oral cavity and lips included 18 studies, accounting for 21 cases (Table 2). Patient ages ranged widely from 9 to 83 years (mean age 49.19±21.45 years; median age 49 years). Among 21 patients, 15 (71.43%) were females, and 6 (28.57%) were males.

**TABLE 2:** Clinical features of unusual oral and labial HSV‐1 infection reported in the literature.

Author/Year	Age	Sex	Location	Clinical presentation	Medication intake	Other diseases	Treatment	Outcome
Alghamdi et al., 2023	69	F	Buccal mucosa, palate, tongue, lips, and cheek skin	Multiple painful ulcers and crusts	Methotrexate and tofacitinib	Rheumatoid arthritis	Oral acyclovir 400 mg, 3x/day ‐ 3 weeks	Complete response
Alsughayer et al., 2024	9	F	Left nostril and upper lip	Large painful ulcer	NA	Immunodeficiency NOS, global developmental delay, epilepsy, hypothyroidism, asthma, plasmablastic lymphoma	IV acyclovir ‐ 14 days	Complete response
Burgoyne et al., 1989	69	M	Anterior dorsal tongue	Multiple painful ulcers	CT—cytosine arabinoside and asparaginase	Acute myeloid leukemia	Oral acyclovir 1000 mg, 1x/day ‐ 5 days	Complete response
	70	F	Anterior dorsal tongue	Single large, firm, violaceous mass	CT—cytosine arabinoside and asparaginase	Acute myeloid leukemia	IV acyclovir 5 mg/kg, 3x/day ‐ 7 days	Complete response
Burke et al., 1994	49	F	Dorsal tongue, tip of tongue, lower lip, palate	Multiple painful ulcers and nodules	Azathioprine, cyclosporin, prednisone, vancomycin, ceftazidime, tobramycin, bumetanide, carafate, and glycopyrrolate	Hepatic failure—the patient underwent an orthotopic liver transplant	IV acyclovir ‐ 2 weeks	Complete response
Hale et al., 1999	69	F	Buccal mucosa, palate, and oropharynx	Multiple small, irregular, painful ulcers	Prednisone	Pemphigus vulgaris	Famciclovir 500 mg ‐ 12 days	Complete response
Ielo et al., 2019	46	F	Dorsal tongue	Painful yellowish serpiginous ulcers	NA	Untreated HIV infection	IV acyclovir 200 mg, 3x/day ‐ 14 weeks	Complete response
Leming et al., 1984	22	F	Dorsal tongue and palate	Painful ulcerated nodules	Vincristine, procarbazine, prednisone, adriamycin, bleomycin, vinblastine, dacarbazine, etoposide, vinblastine, cytosine, and cisplatin	Nodular Sclerosing Hodgkin lymphoma	No treatment	Died of progressive respiratory failure
Lopes et al., 2023	9	F	Soft palate and border of the tongue	Large painful ulcers	CT—ondasetron, vincristine, daunoblastine and L‐asparginase	Acute lymphocytic leukemia	Oral acyclovir 400 mg, 5x/day ‐ 7 days	Complete response
Redding et al., 1990	36	M	Buccal mucosa and border of the tongue	Multiple crateriform painful ulcers	Captopril, lasix, nitroprusside, antithymocyte globulin, cyclosporine, azathioprine, and prednisone	Viral cardiomyopathy—the patient underwent a cardiac transplant	Oral acyclovir 200 mg, 5x/day ‐ 7 days	Complete response
Saunsbury et al., 2021	26	F	Buccal mucosa, palate, retromolar trigone, gingiva, and lips	Large multiple ulcers	Cyclophosphamide and TBI myeloablative conditioning	Myelodysplastic syndrome—the patient underwent allogeneic hematopoietic stem cell transplant	IV acyclovir 10 mg/kg, 3x/day ‐ 10 days, oral valacyclovir 500 mg, 2x/day, IV cidofovir 375 mg 1x/week ‐ 4 weeks	Complete response
Schepanski et al., 2021	40	M	Buccal mucosa, tongue, palate, and commissure	Multiple asymptomatic ulcerated nodules	Nilotinib, cyclosporine, prednisone, levothyroxine, and sulfamethoxazole‐trimethoprim	Acute lymphocytic leukemia	Oral valgancyclovir 1800 mg/day ‐ 30 days	Complete response
Silva et al., 2021a	33	M	Dorsal and ventral tongue and lips	Multiple painful ulcers	The patient is an IV drug abuser	No	IV acyclovir 5 mg/kg, 3x/day	Complete response
Tabaee et al., 2003	48	F	Border of the tongue	Large exophytic mass	Prednisone, tacrolimus, and mycophenolate	Cardiac failure—the patient underwent a cardiac transplant	IV acyclovir 350 mg, 2x/day ‐ 4 days, oral acyclovir 400 mg, 2x/day ‐ 6 days	Complete response
Villa et al., 2013	57	F	Buccal mucosa, palate, and gingiva	Large ulcers	Albuterol, lorazepam, labetalol, lisinopril, moxifloxacin, paroxetine, intravenous immunoglobulin	Common variable immunodeficiency	Oral valacyclovir 500 mg, topical acyclovir 5% 4x/day ‐ 2 months	Complete response
Wicaksono et al., 2024	62	F	Lip commissure	Ulcerated nodule	No	No	Oral acyclovir 400 mg, 5x/day ‐ 7 days	Complete response
Yeom et al., 2023	83	F	Ventral and border of the tongue	Multiple ulcers	Remdesivir and dexamethasone	COVID‐19	NA	NA
	70	F	Dorsal and border of the tongue and lip	Multiple ulcers	Remdesivir, dexamethasone, and methylprednisolone	COVID‐19	NA	NA
	75	M	Lip mucosa and anterior tongue	Multiple ulcers	Remdesivir and dexamethasone	COVID‐19, end‐stage renal disease, and lung carcinoma	NA	NA
Yiu et al., 2019	40	F	Lips and tongue	Multiple black ulcers	Methylprednisolone, basiliximab, tacrolimus, and mycophenolate	The patient underwent a liver transplant	IV acyclovir 600 mg, 14 days	Complete response
Zhao et al., 2025	51	M	Ventral tongue	Small painful nodule	Sirolumus	Von Hippel‐Lindau disease. The patient underwent a kidney transplant	Valacyclovir 1 g daily ‐ 1 month	Complete response

Abbreviations: CT, chemotherapy; IV, intravenous; NA, not available; NOS, not otherwise specified; x, times.

Clinically, most lesions were multiple (*n* = 19; 85.71%) and involved various sites, including the buccal mucosa, tongue, lips, palate, and gingiva. Lesions predominantly presented as ulcers of variable sizes (*n* = 21; 76.19%) or nodular masses (*n* = 5; 23.81%). Immunosuppression was confirmed in most patients, attributed to medication use and comorbidities such as oncologic treatment, transplant history, rheumatologic disorders, infectious diseases, and syndromic conditions. Only a few cases (*n* = 2; 9.52%) represented patients with no apparent immunosuppression.

Patient treatment involved antiviral medications administered at varying dosages. Most patients received oral or intravenous antivirals, including acyclovir, valacyclovir, famciclovir, and valganciclovir, with the majority of cases responding successfully to pharmacological therapy.

Based on the literature review and the findings from this case series, we propose a diagnostic flowchart to assist in the identification of recurrent oral and labial herpes simplex, particularly in atypical or unusual presentations (Figure S1).

## 4 Discussion

This study expands the clinical spectrum of HSV‐1 infection by presenting a comprehensive case series of unusual presentations in both immunocompromised and apparently immunocompetent patients. All cases were classified as unusual due to involvement of non‐keratinized mucosal sites, prolonged clinical course, lesion number (single lesions), large lesion sizes, or unexpected lesion morphology, such as plaque‐like presentations.

The hard palate and attached gingiva are the most common sites for recurrent intraoral herpes infection, likely due to their keratinized mucosal lining [9, 30]. In immunocompromised patients, however, this site‐specific preference disappears, as both keratinized and non‐keratinized mucosa are equally susceptible to infection [7, 9]. Our findings challenge this conventional distribution even in immunocompetent individuals. In our cohort, lesions most frequently involved the tongue border (50% of cases), and the majority arose in non‐keratinized locations. This prevalence is strikingly higher than the 7.7% (4/52) rate of non‐keratinized site involvement reported in a large immunocompetent cohort [30]. This stark contrast not only underscores the highly unusual nature of our case series but also provides a clear explanation for the high rate of initial misdiagnosis observed in our study. Since the lateral tongue is an uncommon site for recurrent HSV‐1, clinicians do not routinely consider it in the differential diagnosis for ulcers in this location, leading to diagnostic delay and inappropriate management, particularly as only one patient in our cohort had a confirmed immunocompromised status. This reinforces that such presentations can and do occur in immunocompetent hosts.

Immunosuppressed patients exhibit deficiencies in cell‐mediated immunity, leading to impaired immune response against HSV‐1 and unusual or atypical clinical manifestations [11]. Interestingly, a study conducted by de Faria et al. [31] observed viral‐induced changes consistent with herpes infection in the tongues of 10 out of 92 cadavers who had died from AIDS and were examined during autopsy, one of which also showed evidence of cytomegalovirus infection.

For the management of patients with unusual clinical presentations, the first critical step is to exclude possible undiagnosed immunosuppression [30]. Our finding that the majority of patients in our series were immunocompetent finds support in the literature, though such reports remain scarce. A recent study described an atypical, nodular labial herpes presentation in a 62‐year‐old immunocompetent woman, which aligns with our observation of unusual presentations occurring without underlying immunosuppression [25]. While the mechanisms driving such manifestations in otherwise healthy individuals remain incompletely explored, factors such as advanced age, poor nutritional status, or stress may induce transient immunosuppression, potentially compromising cellular immunity and enabling unusual presentations [32]. The accumulating evidence from our series and these rare reports underscores that a lack of overt immunosuppression does not rule out an atypical HSV‐1 infection, challenging the prevailing clinical paradigm. Notably, an immunocompromised background in a sole patient in this cohort was explained by a methotrexate and immunotherapy‐based regimen, similar to a prior reported case [15].

Herpetic infection diagnosis typically relies on clinical history and intraoral examination [7]. However, in unusual cases, laboratory testing using PCR, cytology, or viral culture may be necessary [30, 33]. In this study, all cases were histopathologically confirmed, as herpes infection was not initially suspected clinically. For unusual lesions, we emphasize the necessity of biopsy followed by histopathological and IHC analysis [14, 24]. Microscopic hallmarks of HSV‐1 infection include typical cytopathic effects in keratinocytes, such as multinucleation, nuclear molding, and glassy nuclei [26]. The microscopic findings characteristic of herpes infection are preferentially observed at the edges of the ulcer, a region characterized by intense epithelial activity aimed at re‐epithelializing the ulcerated area. Therefore, pathologists must pay close attention to the margins of the lesion, where these morphological features tend to be more evident.

Eventually, infected keratinocytes may be obscured by dense inflammatory infiltrate, making IHC evaluation using HSV‐1‐specific antibodies essential for accurate diagnosis [14, 22]. Since PCR results may occasionally be negative, histopathology remains a critical diagnostic approach and a pivotal step in ensuring accurate diagnosis and effective clinical management [16].

The management of recurrent HSV‐1 infection in immunocompetent individuals focuses primarily on symptomatic relief through analgesics and anti‐inflammatory agents. However, topical or systemic antiviral therapy (e.g., acyclovir, valacyclovir) may significantly accelerate lesion resolution and improve clinical outcomes [33]. This is particularly relevant for unusual presentations, where empiric antiviral administration may be warranted to exclude herpes infection [24]. In immunocompromised patients, treatment resistance is not uncommon, often necessitating alternative antiviral regimens and combined treatment approaches [22]. Our findings corroborate the utility of multimodal therapy. In case #2, photobiomodulation reduced lesion size, but complete resolution required subsequent antiviral treatment. This aligns with evidence that photobiomodulation serves as a valuable adjuvant for intraoral HSV‐1, typically showing efficacy in small lesions [34], but may be insufficient as monotherapy for larger or persistent atypical presentations. Thus, while adjuvant therapies provide benefit, antiviral treatment remains central to managing complex cases.

This study has limitations inherent to its retrospective, case‐series design, including a small sample size and the absence of universal PCR confirmation. While these factors reflect the rarity of the condition, they preclude prevalence calculations and subgroup analyses. Future prospective, multicenter studies are needed to validate our findings, and molecular investigations should explore potential viral or immune mechanisms underlying these unusual presentations in immunocompetent hosts.

## 5 Conclusion

Our findings highlighted that immunocompetent patients may exhibit unusual clinical presentations of HSV‐1 infection. These lesions can manifest as solitary, painful ulcers or white plaque‐like lesions involving either keratinized or non‐keratinized mucosa. Histopathological evaluation remains critical for accurate diagnosis and to guide appropriate therapeutic strategies. Clinical management of unusual HSV‐1 infection should prioritize ruling out undiagnosed immunosuppression and administering antiviral therapy to optimize clinical outcomes. Clinicians and pathologists must be aware of these uncommon manifestations of oral herpes.

## Author Contributions

**João Paulo Gonçalves de Paiva**: writing – original draft, data curation. **Sebastião Silvério Sousa‐Neto**: writing – original draft, data curation. **Maurilo Antônio Correia Humberto**: writing – review & editing, data curation. **Marco Antônio de Oliveira** magalhães: writing – review & editing, data curation. **Justin Bubola**: writing – review & editing, data curation. **José Narciso Rosa Assunção Júnior**: writing – review & editing, data curation. **Marcelo Marcucci**: writing – review & editing, data curation. **Marcondes Sena‐Filho**: Writing – review & editing, Data curation. **Celso Augusto Lemos**: Writing – review & editing, Data curation. **Jacks Jorge**: Writing – review & editing, Methodology, Formal analysis. **Pablo Agustin Vargas**: Writing – review & editing, Methodology, Formal analysis.

## Funding

This study was partially financed by Coordenação de Aperfeiçoamento de Pessoal de Nível Superior (CAPES) and São Paulo Research Foundation (FAPESP). João Paulo Gonçalves de Paiva and Sebastião Silvério Sousa‐Neto are Doctoral student researchers who hold a fellowship funded by CAPES (Finance Code 001) and FAPESP (2023/13797‐6), respectively.

Informed consent was obtained from all individual participants in this study.

## Disclosure

All procedures performed in studies involving human participants were in accordance with the ethical standards of the institutional and/or national research committee (Piracicaba Dental School Ethics Research Committee; CAAE: 89601925.4.0000.5418) and with the 1964 Helsinki Declaration and its later amendments or comparable ethical standards.

During the preparation of this work, the author(s) used ChatGPT (GPT‐4 model, OpenAI) in order to improve the readability and language of the manuscript. After using this tool/service, the authors reviewed and edited the content as needed and takes full responsibility for the content of the published article.

## Consent for Publication

Consent for publication was obtained for every individual person's data included in this study.

## Conflicts of Interest

The authors declare no conflicts of interest.

## Supporting information




**Supplementary Figure 1**. Flowchart proposed for the diagnosis of oral HSV‐1 infections, including atypical presentations. The diagram highlights the importance of clinical history, lesion persistence, and morphological evaluation, especially cellular changes suggestive of herpes infection at the edges of ulcerated lesions. Ancillary tests, such as immunohistochemistry for HSV‐1, are recommended in inconclusive cases to support the diagnosis and guide therapeutic decisions.

## Data Availability

The authors have nothing to report.
