# Supplementary figures and images for: Unusual Oral Presentations of Herpes Simplex Virus‐1: A Series of Eight Cases and Literature Review
Source: Spec Care Dentist. 2025 Dec 18;45(6):e70129. doi: 10.1111/scd.70129 (PMC12715298; doi:10.1111/scd.70129)

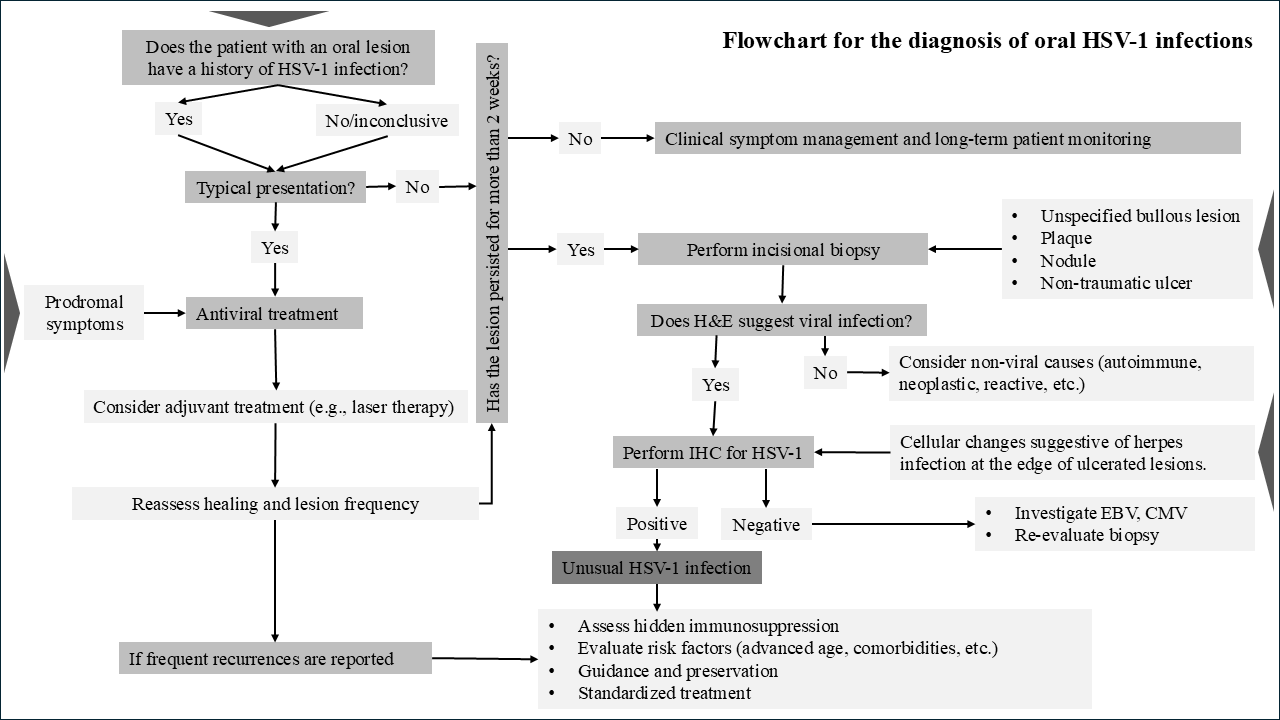

Supplement: Supplementary file 1 — Supplementary Figure 1. Flowchart proposed for the diagnosis of oral HSV‐1 infections, including atypical presentations. The diagram highlights the importance of clinical history, lesion persistence, and morphological evaluation, especially cellular changes suggestive of herpes infection at the edges of ulcerated lesions. Ancillary tests, such as immunohistochemistry for HSV‐1, are recommended in inconclusive cases to support the diagnosis and guide therapeutic decisions. [file SCD-45-0-s001.tif]
